# The association between muscle architecture and muscle spindle abundance

**DOI:** 10.1038/s41598-023-30044-w

**Published:** 2023-02-17

**Authors:** Roger W. P. Kissane, James P. Charles, Robert W. Banks, Karl T. Bates

**Affiliations:** 1 Department of Musculoskeletal & Ageing Science, Institute of Life Course & Medical Science, University of Liverpool, The William Henry Duncan Building, 6 West Derby Street, Liverpool, L7 8TX UK; 2 Department of Biosciences and Biophysical Sciences Institute, University of Durham, South Road, Durham, DH1 3LE UK

**Keywords:** Skeletal muscle, Somatic system

## Abstract

Across the human body, skeletal muscles have a broad range of biomechanical roles that employ complex proprioceptive control strategies to successfully execute a desired movement. This information is derived from peripherally located sensory apparatus, the muscle spindle and Golgi tendon organs. The abundance of these sensory organs, particularly muscle spindles, is known to differ considerably across individual muscles. Here we present a comprehensive data set of 119 muscles across the human body including architectural properties (muscle fibre length, mass, pennation angle and physiological cross-sectional area) and statistically test their relationships with absolute spindle number and relative spindle abundance (the residual value of the linear regression of the log-transformed spindle number and muscle mass). These data highlight a significant positive relationship between muscle spindle number and fibre length, emphasising the importance of fibre length as an input into the central nervous system. However, there appears to be no relationship between muscles architecturally optimised to function as displacement specialists and their provision of muscle spindles. Additionally, while there appears to be regional differences in muscle spindle abundance, independent of muscle mass and fibre length, our data provide no support for the hypothesis that muscle spindle abundance is related to anatomical specialisation.

## Introduction

Skeletal muscles are comprised of two functionally distinct types of muscle fibre: extrafusal fibres, whose primary role is to generate power and movement; and the intrafusal fibres of muscle spindles, whose principal function is to modify the sensory endings’ responses to changes in muscle length^1,2^. Within a single organism, muscles exhibit diverse biomechanical roles, from the large quadriceps and hamstring muscles integral for walking^3^ to the smaller muscles of the eye which function to produce rapid eye movements and stabilisation^4^. It is a commonly held belief that the provision of muscle spindles reflects the functional demands of a given muscle^5–8^, with some hypothesising that muscles with high spindle densities (number of muscle spindles per gram) are primarily involved in fine motor control^5^ or function as kinesiological sensors^6^. There are, however, several fundamental issues with this hypothesis. The first major issue is the use of spindle density to quantify the abundance of spindles within skeletal muscle. Spindle density exhibits a non-linear relationship with muscle mass^9–11^ making it highly misleading to infer linear comparisons with muscles of different sizes. Instead, any inference of spindle provision should be based on a suitably transformed linear relationship^12^. Subsequently, the residual value of the linear regression of the log-transformed spindle number and muscle mass as an unbiased measure has become the prevailing descriptor of spindle abundance^10,11,13^. Residual values are homogenously distributed, allow for back-transformation calculations of spindle number, and provide the most unbiased comparative measure of relative spindle abundance^10,11^. The second problem with this hypothesis is the general lack of quantitative data defining muscle function, which is often described in subjective qualitative terms such as ‘fine motor control’.

Through the novel application of musculoskeletal modelling of human walking, it has been shown that muscles of the leg considered to be highly abundant in muscle spindles tend to function more like springs, while those less abundant typically functioned more as brakes during overground walking ^13^. This provides the first quantitative insight into the potential physiological determinants of muscle spindle abundance. However, muscle spindle abundance not only varies within a single locomotor muscle group but also between muscle groups, where for example muscle spindles are significantly more abundant in axial muscles and those of the neck compared to those of arm, legs, hands and feet^10^. This may be indicative of distinct biomechanical roles or control strategies between anatomical regions^10,11^. Thus, despite the significant correlation of muscle architecture (muscle fibre length and pennation) with muscle spindle number of the leg^13^, it cannot be assumed to hold true across the entire human body. Therefore, in this study, we have collated a comprehensive data set of human muscles (119 muscles across nine body regions) describing muscle architecture to test correlations between muscle spindle abundance and muscle anatomy, with the aim of investigating the relationships between muscle architecture, spindle number and spindle abundance.

Firstly, given the perception of muscle spindles being primarily length sensors^10,14–17^ and the findings in the leg muscle^13^ we hypothesise that muscle spindle provision will correlate with anatomical derivatives important to muscle length change across the entire human body (e.g. muscle fibre length and pennation angle), Hypothesis 1. Secondly, using morphospace plots of extrafusal fibre length and PCSA we will determine whether muscles with greater abundances of muscle spindles have architectural properties optimised for function as displacement specialists^18–21^ as is often hypothesised, Hypothesis 2^5–7^. Finally, we test to see if there are differences in functional specialisation across body regions that may account for the heterogeneity in spindle abundance across the body^10^, Hypothesis 3.

## Results

### Architectural correlates of muscle spindle provision (Hypothesis 1–2)

**Figure 1:**
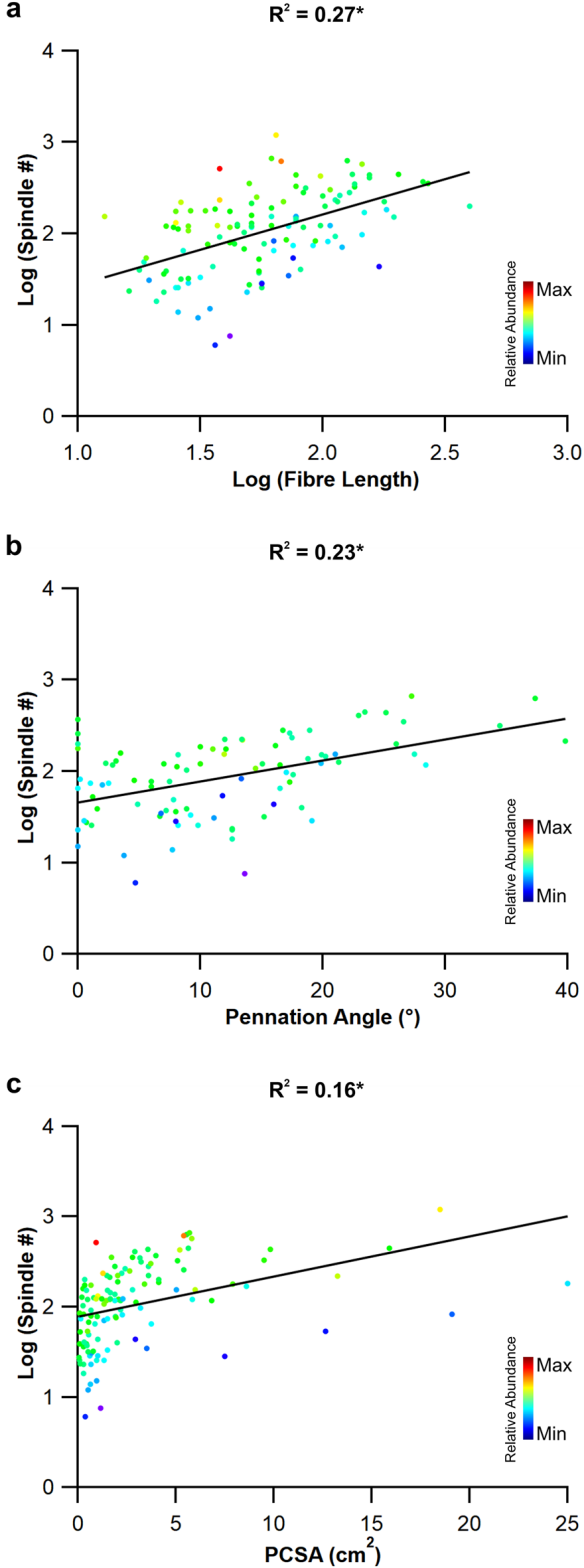
Muscle spindle number correlates with fibre length, muscle pennation and physiological cross-sectional area. Spindle number is correlated with muscle fibre length (**a**), muscle pennation (**b**) and muscle physiological cross-sectional area (**c**). Muscle spindle abundance as indicated by the heatmap highlights that neither fibre length, muscle pennation nor physiological cross-sectional area correlated with muscle spindle abundance. *P < 0.05.

**Figure 2:**
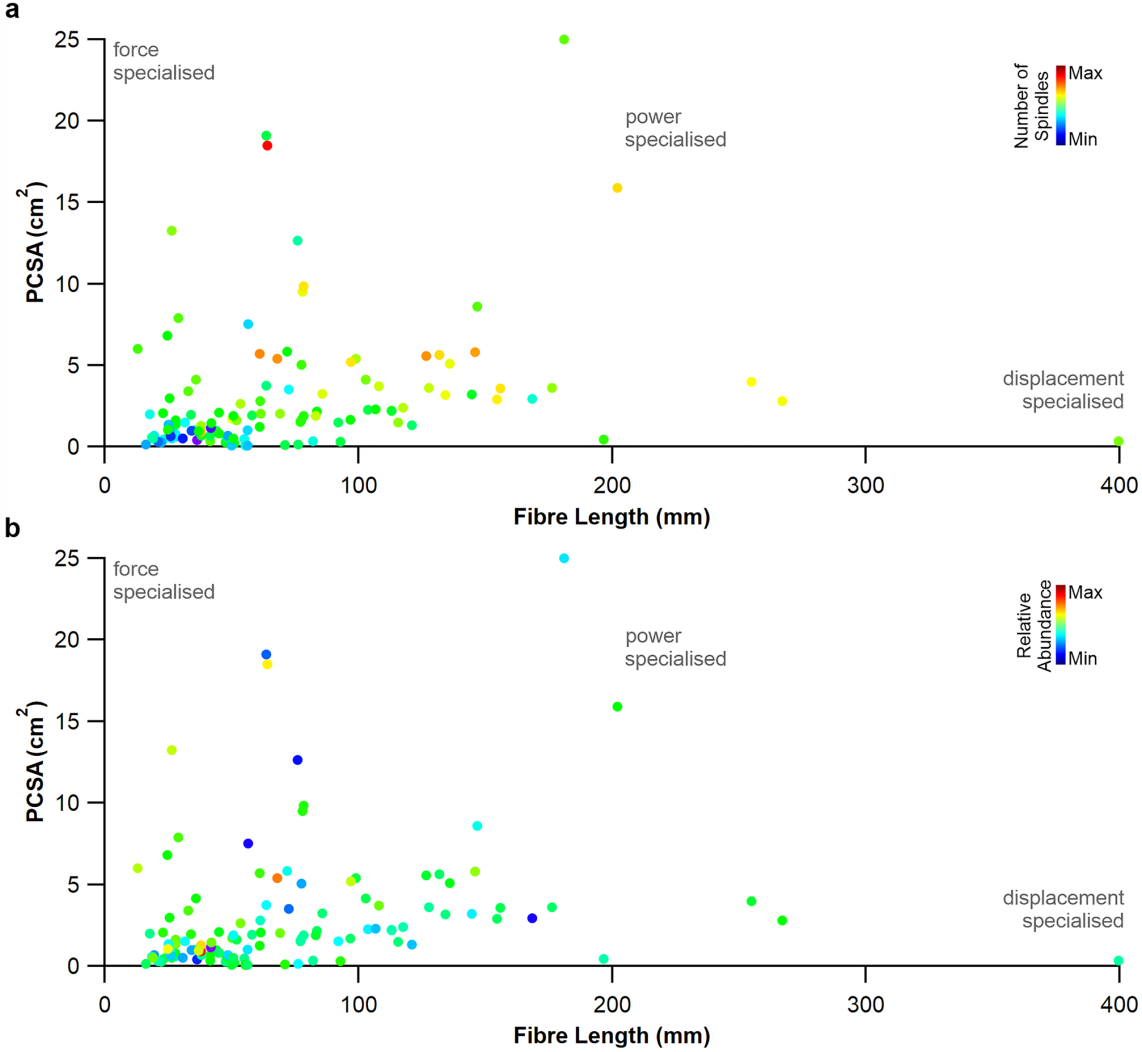
Morphospace plots show no correlation between muscle spindle composition with displacement specialists. Muscles that neither contain large absolute numbers of muscle spindles (**a**) nor are highly abundant in spindles (**b**) appeared to be preferentially optimised as displacement specialists.

Across all 119 muscles tested, muscle fibre length (R^2^ = 0.27, *P* < 0.001, Fig. 1a), pennation angle (R^2^ = 0.23, *P* < 0.001, Fig. 1b) and PCSA (R^2^ = 0.16, *P* < 0.001, Fig. 1c) all presented a significant positive correlation with muscle spindle number. There were, however, no correlations with relative spindle abundance and fibre length (R^2^ = 0.024; *P* = 0.06; Fig. 1a, Supplementary Fig. 2a), pennation angle (R^2^ = 0.001; *P* = 0.36; Fig. 1b, Supplementary Fig. 2b) or PCSA (R^2^ = 0.005, *P* = 0.16, Fig. 1c, Supplementary Fig. 2c). The functional morphospace plots show that across the 119 human muscles there was no clear grouping of muscles based on spindle number (Fig. 2a, Supplemental Fig. 3) or spindle abundance (Fig. 2b, Supplemental Fig. 4) with those architecturally optimised to function as displacement specialists (i.e. long muscle fibre length and low PCSA).

**Figure 3:**
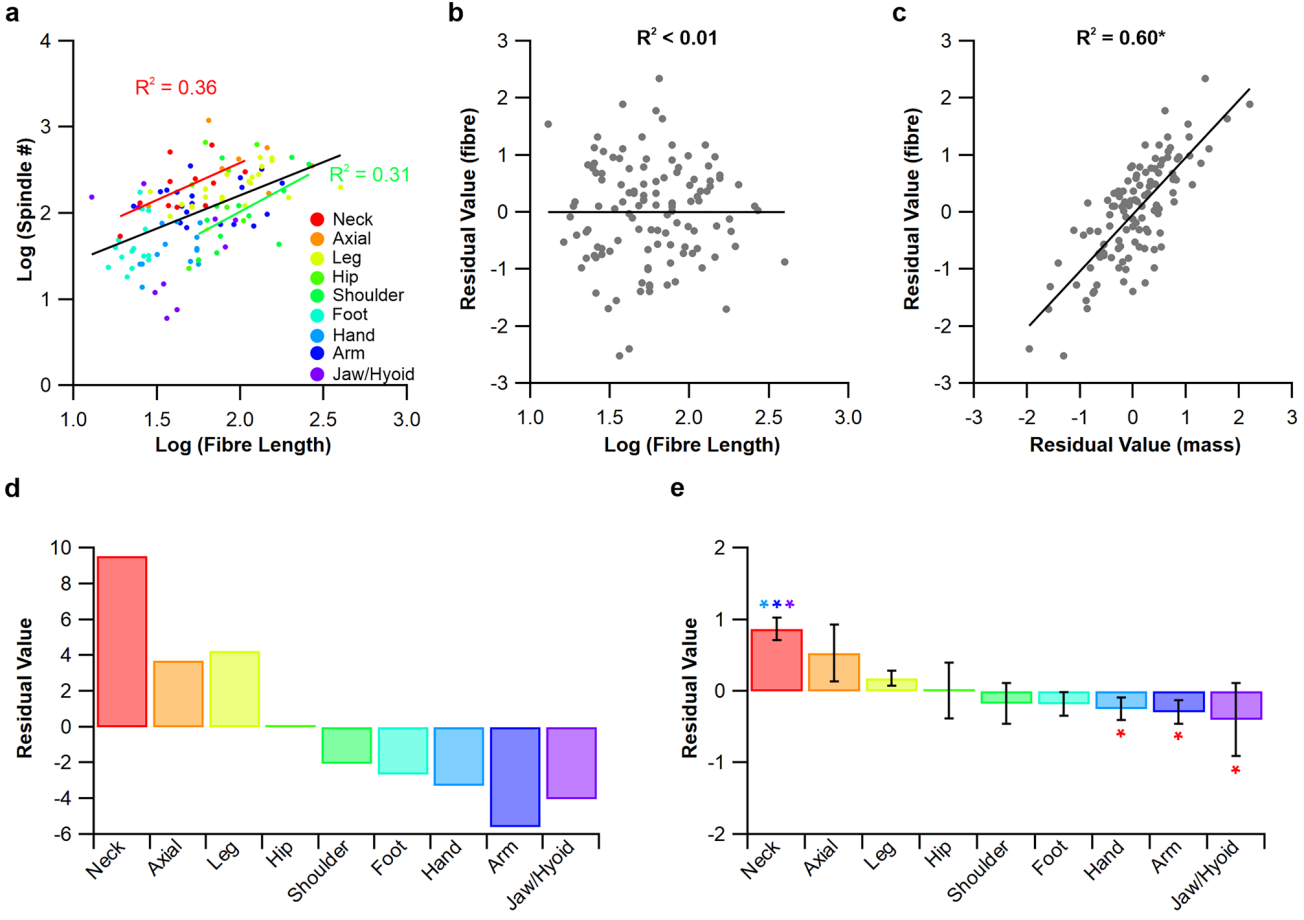
Regional differences in spindle composition. Muscles have been grouped into regions of the body and linear regressions plotted for the neck (red line) and shoulder (green line) (**a**). These data follow an identical trend to those of Banks (2006) where muscles in the neck appear to be more highly abundant in muscle spindles compared to those of the shoulder. Using the grouped data linear regression (black line) residual values have been calculated for each muscle (**b**). Taking the residual values estimated by muscle mass from Banks (2006) and those calculated here for fibre length we show that there exists a significant overlap between the two residual value methods (**c**). This highlights the tightly coupled relationship between muscle spindle number and muscle architecture, while providing further support for the use of residual values as a measure of muscle spindle abundance. The sum (**d**) and average (**e**) residual value across regions highlight the difference in abundance across the body. *P < 0.05.

**Figure 4:**
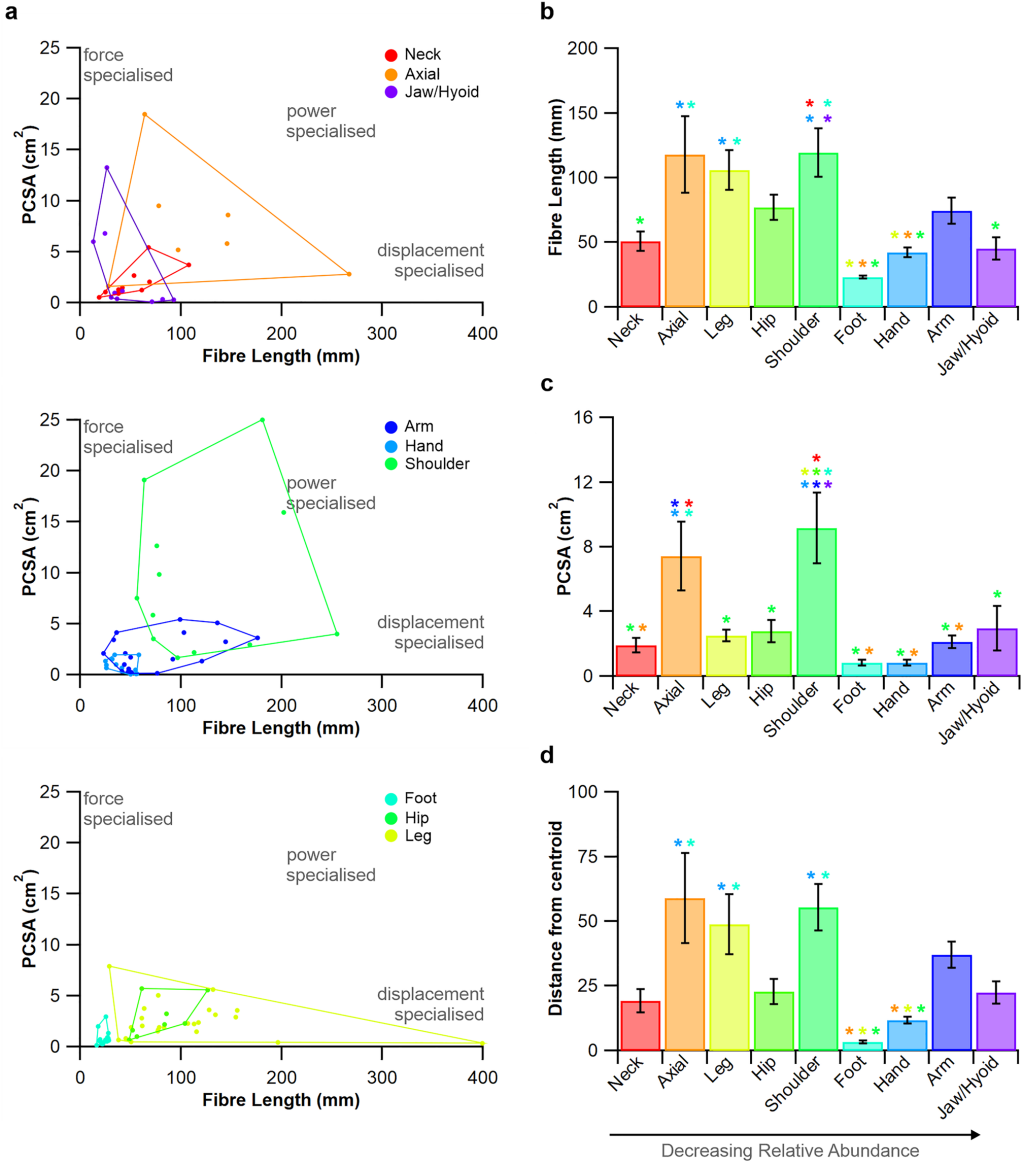
Morphospace plots of regional body parts. To discern potential anatomical parameters that might underpin such regional differences in spindle abundance we have plotted muscle architectural parameters for each of the nine body regions (**a**). Averaged muscle fibre lengths (**b**) PCSA (**c**) and architectural disparity: the mean distance of each individual muscle from the centroid of the outlined area (**d**). *P < 0.05.

### Heterogeneity in muscle architecture across body regions (Hypothesis 3)

When looking at the distribution of muscle spindle abundance across the newly realised correlate of muscle fibre length and muscle spindle number (Fig. 1a), muscles with a greater relative abundance appear to sit above the regression line, and those with a lower relative abundance appear to sit below this line. We find that muscles in the neck sit above this relationship, while those of the shoulder sit below it (Fig. 3a), consistent with the pattern seen when plotting muscle mass against muscle spindle number^10^. This suggests there exist regional differences in relative spindle abundance when calculated from the relationship between muscle spindle number and fibre length. Therefore, through the generation of fibre length-derived residual values (Fig. 3b) we show that a significant positive relationship exists with those estimated using muscle mass (R^2^ = 0.60, *P* < 0.001, Fig. 3c) and that regional differences exist in muscle spindle abundance when derived from fibre length (Fig. 3d,e). Individual body regions occupied distinct areas across the morphospace plots (Fig. 4a), with significant regional differences in muscle fibre length (F(8) = 5.864, *P* < 0.001, Fig. 4b) and PCSA (F(8) = 8.413, *P* < 0.001, Fig. 4c). There appeared to be no relationship with relative spindle abundance. Finally, anatomical disparity (F(8) = 5.402, *P* < 0.001, Fig. 4d) significantly varied across body segments and appeared to show no underpinning relationship with muscle spindle abundance.

## Discussion

There has been a long-held belief that muscle spindles function primarily as length and velocity sensors and that functional specialisation underpins the number of muscle spindles per unit of mass^17,22^. Recent work has shown that muscle spindle number across the muscles of the leg significantly correlates with muscle fibre length. Additionally, there appears to be a strong association between muscle spindle abundance and the biomechanical function of the muscles during walking^13^. Yet, we still lack an understanding of whether such anatomical correlates hold true across multiple body regions that undergo different locomotor behaviours and central control strategies^10,11,13^. Through the compilation of the most comprehensive architectural dataset of human skeletal muscle, we have uncovered novel correlates with muscle spindle provision. Here we show that across all body regions that absolute muscle spindle number correlates not only with muscle mass but also muscle fibre length, pennation angle and PCSA (Hypothesis 1). Additionally, we find no support for the commonly held belief that muscles optimised to function as displacement specialists have a greater muscle spindle abundance (Hypothesis 2). Finally, we show that muscle spindle abundances derived from fibre length mirror those generated from muscle mass, and subsequently highlight the complexity of muscle architecture and muscle spindle abundance (Hypothesis 3).

### Anatomical correlates with muscle spindle composition

Here we show that muscle spindle number is significantly related to muscle fibre length, fibre pennation angle and PCSA (Fig. 1). Despite this significant relationship, there does not appear to be any relationship with muscle spindle abundance (Fig. 1, Supplementary Fig. 2). It has long been thought that muscle fibre length is a key input signal to the central nervous system^17,22^ and the correlations shown here provide statistical evidence of this, thus supporting our first hypothesis. The functional capacity of skeletal muscle is highly influenced by its architectural properties^21^, where muscles optimised to generate force are composed of short fibre lengths and large PCSA, compared to muscles specialised to undergo large strain amplitudes that are typically composed of long fibre and small PCSA^18–21,23–25^. It has long been thought that muscles containing a greater number of spindles per unit of mass are specialised to function as displacement specialists or as kinesiological sensors^5,6^ without any reliable measure of muscle function having been made. We have recently shown that within the muscles of the human lower limb those exhibiting greater absolute numbers of spindles or their relative abundance are not those architecturally optimised as displacement specialists^13^. Our data here provides evidence that this is consistent across individual muscle groups of the human musculoskeletal system (Supplemental Figs. 1 and 2), with muscles containing a greater provision of spindles not necessarily being those optimised to function as displacement specialists, thus rejecting hypothesis 2.

### Regional differences in muscle spindle abundance

Having identified the significant relationship between muscle spindle number and fibre length, the logical next step was to calculate the residual values of this relationship to see if equivalent regional differences existed to those estimated by Banks^10^. Subsequently, we show that muscle spindle abundance estimated by muscle fibre length-derived residual values (Fig. 3) significantly overlap with those estimated by muscle mass^10^, with, for example, muscles in the neck being significantly more abundant in spindles than those of the hand or arm (Fig. 3d,e). By using the residual value, their homogeneous distribution with respect to fibre length (Fig. 3b) and mass^10^ provides a systematic and unbiased method to compare the relative abundance of muscle spindle^10,11,13^, where previous measures like spindle density are sensitive to increases in muscle size. Given the lack of correlation between muscle fibre length (Fig. 1a), pennation angle (Fig. 1b) and PCSA (Fig. 2b) with relative spindle abundance, it is perhaps not surprising that regional differences in muscle spindle abundance are not predicted by muscle architecture (Fig. 4). These data suggest that no single anatomical correlate with relative spindle abundance exists across the human body, expanding upon previous work limited only to muscles of the leg^13^ (Hypothesis 3).

There exists an inherent difficulty in uncovering anatomical and functional relationships with muscle spindle provision. This is in part due to the methods of quantification, where data are primarily derived from serial transverse histological sections from human cadaveric preparations which constrains the generation of additional morphometric indices (e.g. muscle fibre length and PCSA). Our novel approach relies on the curation of anatomical data, which we know to be highly variable, especially fibre length which is especially susceptible to error^26^. Additionally, while there is variability across the masses between our two data sets (Supplementary Table 1), the similarities in residual values between those derived from mass and from fibre length are encouraging and would in our opinion unlikely be a result of variability in the data. Despite the development of fluorescently labelled proprioceptive sensory endings in mice^27,28^ the relative abundances of muscle spindles within the common laboratory mouse are still unknown for all but a few muscles. Therefore, to further progress our knowledge of physiological determinants of muscle spindle abundance we rely on using the only comprehensive data set spanning 137 human muscles^10^. Through musculoskeletal modelling and simulation of human walking, we recovered novel findings suggesting muscle spindle abundance is underpinned by gross in vivo function^13^, paving the way for comparable experimentation to be done across body regions (e.g. head/neck^29^ *vs.* arm/hand^30^) to explore the biomechanical underpinning of heterogeneity in muscle spindle abundance.

## Conclusion

Through the assembly of this comprehensive anatomical data set, we have uncovered novel correlates of muscle architecture and muscle spindle provision. We find no statistical support for the idea that muscles containing larger numbers of muscle spindles are those optimised to function as displacement specialists. Critically, our approach extends on from previous work and has uncovered that there exists a tightly coupled relationship between muscle fibre length and spindle number. These data further emphasise the importance of muscle fibre length as a critical input into the central nervous system, while also highlighting the complexity of physiological determinants of muscle spindle abundance.

## Methods

### Published sources

**Figure 5:**
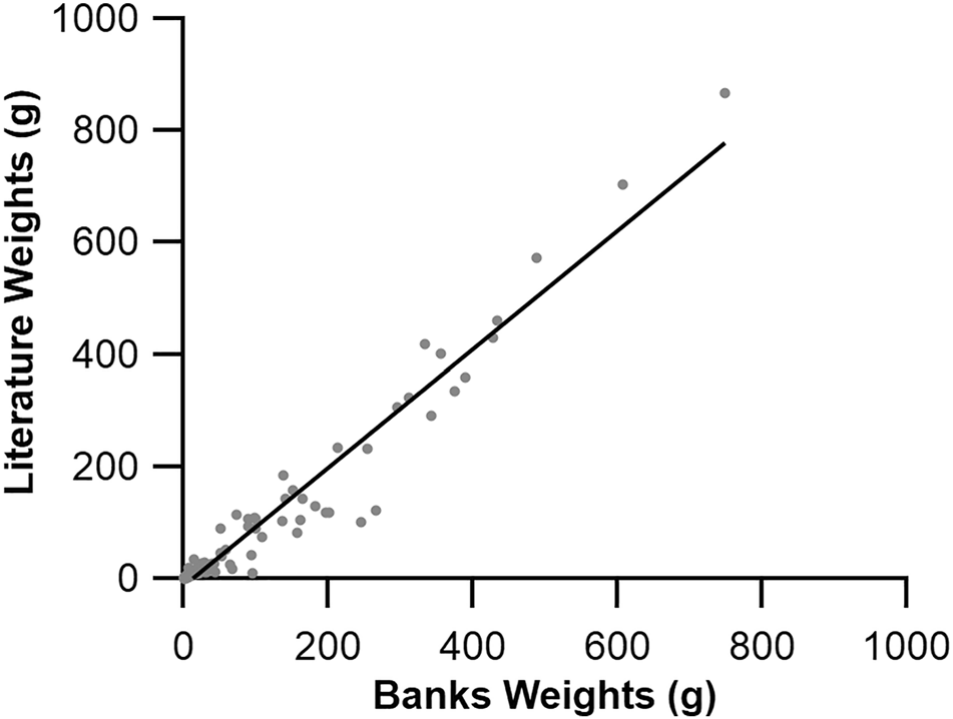
Comparison of muscle masses. Comparison of human muscle masses taken from Banks (2006) and comparative muscle architecture publications (see ESM1). R^2^ = 0.95, *P* < 0.0001.

Absolute muscle spindle counts, relative abundance and muscle weights were taken from Banks^10^. Absolute muscle spindle counts were originally derived from serial cross-sections of muscle and describes the number of separately identifiable capsular expansions containing sensory innervation^31^. Relative abundance of muscle spindles is calculated as the residual value of the linear regression of the log-transformed spindle number and muscle mass were taken directly from Banks (2006) Appendix 1. Muscle architecture data were compiled from multiple sources^32–49^ (see ESM 1). Muscle fibre lengths (L_f_) and physiological cross-sectional areas (PCSA) for 119 muscles were collated from human specimens whose muscle mass was near identical to the samples from Banks^10^ (R^2^ = 0.95, *P* < 0.001, Fig. 5). Individual muscle masses showed there to be a strong significant relationship across the axial, hand, hip, hyoid/jaw, leg, neck and shoulder (Supplementary Fig. 1, Supplementary Table 1), while the arm and foot were not as strongly related. Given that the number of muscle spindles is considered to be fixed at birth^10,50^, these subtle differences in the arm and foot muscles are unlikely to impact the conclusions drawn from the work here.

### Original data

Nine subjects were recruited (4 Male, 5 Female; Age: 29 ± 3 years; Body mass: 68 ± 10 kg; Height: 175 ± 8 cm; BMI: 21.9 ± 1.8 kgm^−2^) who provided informed consent prior to participating in the study in accordance with ethical approval from the University of Liverpool’s Central University Research Ethics Committee for Physical Interventions (Reference number: 3757). This work was conducted in accordance with the declaration of Helsinki and the regulations set out within the ethical guidelines and that of the journal.

Foot muscle architecture data from 5 muscles of the right foot were collected from each subject, as previously described^51^. Briefly, this involved a T1-weighted anatomical turbo spin echo (TSE) MRI sequence to estimate muscle volumes and visualise muscle attachment points. Muscle fibre length for the flexor digitorum brevis, flexor hallucis brevis, abductor hallucis, abductor digiti minimi pedis and adductor hallucis (ESM2) we estimated from muscle belly length using correction factors for muscle fibre length:muscle length^52^ and pennation angle^53^.

### Data analysis

Given the role of muscle spindles as length sensors, we expect there to be a strong relationship between muscle spindle provision and measures affecting muscle length change, namely fibre length and pennation angle (Hypothesis 1) akin to that seen in the leg muscles of humans^13^. Model II simple regressions (reduced major axis) were therefore conducted to tests for significant linear relationships between these muscle architecture metrics and spindle composition (absolute spindle number and relative spindle abundance, taken from^10^) and muscle architecture (fibre length and fibre pennation). Using scatter plots of fibre length and PCSA we examine the architectural specialisation of individual muscles^18–21,23–25^. Muscles with long fibre lengths and low PCSA were classed as displacement specialised, long L_f_ and high PCSA as power specialised and short L_f_ and high PCSA as force specialised. Here we use the morphospace plots to examine whether muscle spindle provision was associated with muscles whose architecture (fibre length and PCSA) are optimised to function as displacement specialists (i.e. long muscle fibre length and low PCSA, Hypothesis 2). Finally, to discern if regional differences in relative spindle abundance were associated with muscle architecture optimised function, muscles were grouped into categories as described in Banks (2006) (arm, axial, foot, hand, hip, hyoid/jaw, leg, neck and shoulder). The variance in muscle architecture across body segments was described by the disparity of individual muscles in morphospace^54^. Briefly, the disparity of individual limbs is calculated as the mean Euclidean distance of all muscles of a given body segment from the centroid (average x–y position of PCSA *vs.* fibre length). To test for regional differences in muscle architecture (fibre length and PCSA) and morphospace disparity one-way analysis of variation (ANOVA) was employed (Hypothesis 3). Where significance was detected post-hoc comparisons were made using the Bonferroni correction. All linear regressions were completed in R using the ‘lmodel2’ package, while the ANOVAs were completed using SPSS (v25), with the threshold for statistical significance set to *P* < 0.05.

## Supplementary Information

Supplementary Information 1.

Supplementary Information 2.

## Data Availability

All data is contained within the electronic supplementary material (ESM).
