# Supplementary material for: The association between muscle architecture and muscle spindle abundance
Source: Sci Rep. 2023 Feb 17;13:2830. doi: 10.1038/s41598-023-30044-w (PMC9938265; doi:10.1038/s41598-023-30044-w)
Supplement: Supplementary file 2 — Supplementary Information 2. [file 41598_2023_30044_MOESM2_ESM.docx]

The association between muscle architecture and muscle spindle abundance

Roger W. P. Kissane^1†*^, James P. Charles^1†^, Robert W. Banks^2^ & Karl T. Bates^1^

^1^ Department of Musculoskeletal & Ageing Science, Institute of Life Course & Medical Science, University of Liverpool, The William Henry Duncan Building, 6 West Derby Street, Liverpool L7 8TX, UK

^2^ Department of Biosciences and Biophysical Sciences Institute, University of Durham, South Road, Durham DH1 3LE, UK

^†^Joint first authors

*Correspondence to: r.kissane@liverpool.ac.uk

**Running Title:** Structural underpinning of muscle spindle abundance

**Key words:** Muscle Spindle, Physiological Cross-Sectional Area, Muscle Architecture,

**Supplementary Figures**


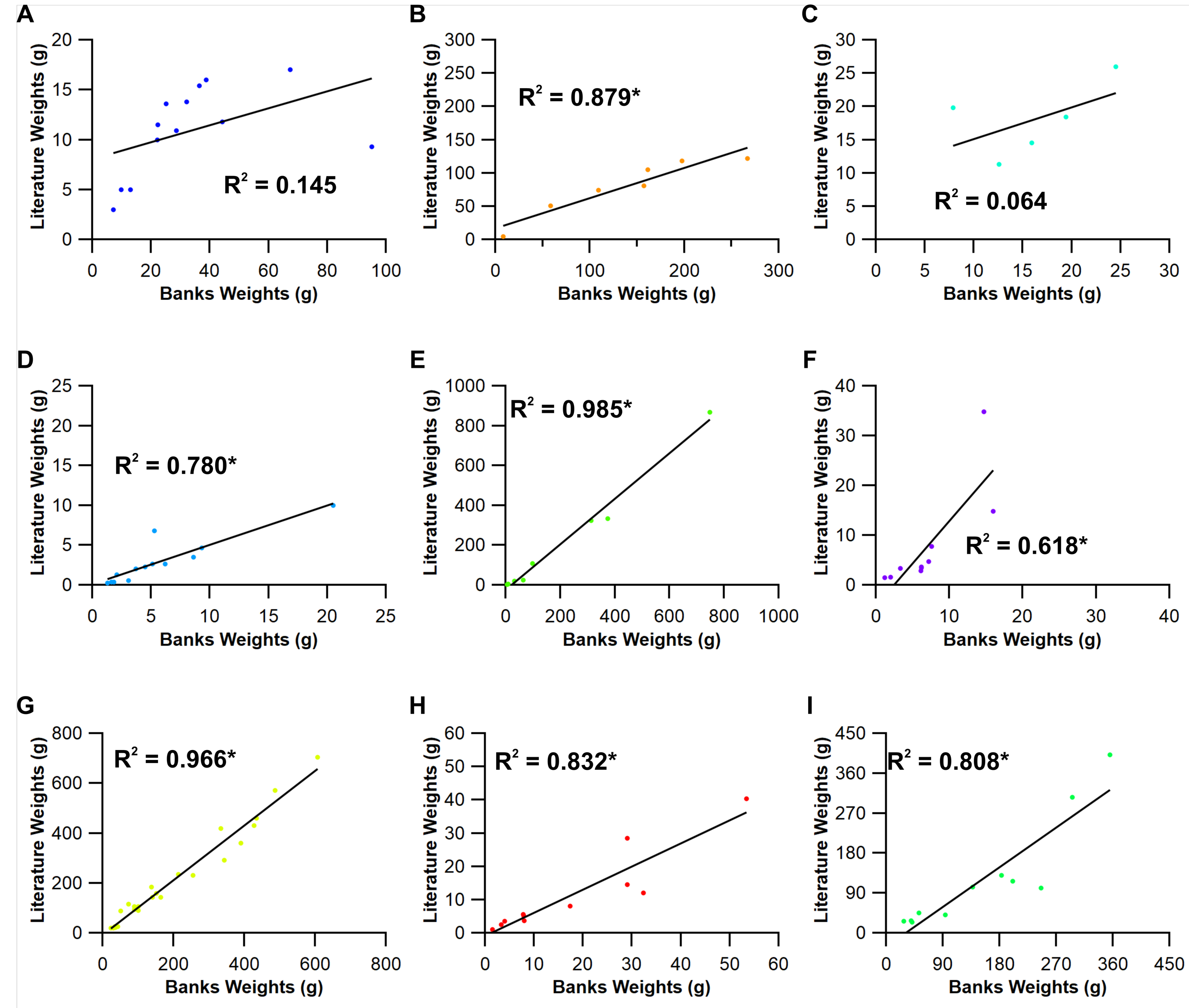


**Supplementary Figure 1. Comparison of muscle masses across body regions.** Comparison of human muscle masses taken from Banks (2006) and comparative muscle architecture publications (see ESM1) for the arm (A), axial (B), foot (C), hand (D), hip, (E), hyoid/jaw (F), leg (G), neck (H) and shoulder (I). * P<0.05.


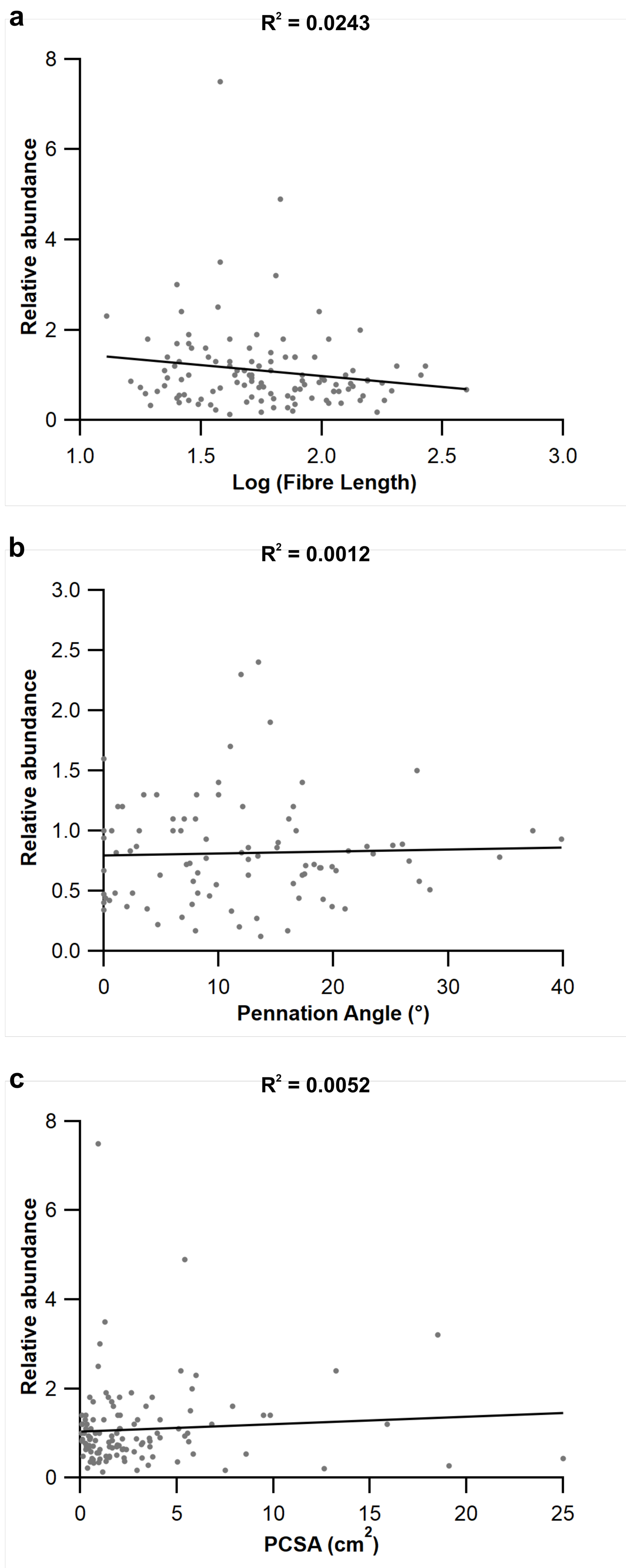


**Figure 2. Relative spindle abundance does not correlates with fibre length, muscle pennation or physiological cross-sectional area.** Relative spindle number is not correlated with muscle fibre length (a), muscle pennation (b) or muscle physiological cross-sectional area (c).

**Supplementary Figure 3. Morphospace plots of regional body parts and spindle number.** To discern potential anatomical parameters that might underpin such regional differences in spindle provision we have plotted muscle architectural parameters for each of the nine body regions: leg (a), jaw/hyoid (b), hand (c), hip (d), axial (e), shoulder (f), foot (g), neck (h) and arm (i). Heatmap colours correspond to the absolute numbers of spindles contained within the muscle.

**Supplementary Figure 4. Morphospace plots of regional body parts and spindle abundance.** To discern potential anatomical parameters that might underpin such regional differences in spindle provision we have plotted muscle architectural parameters for each of the nine body regions: leg (a), jaw/hyoid (b), hand (c), hip (d), axial (e), shoulder (f), foot (g), neck (h) and arm (i). Heatmap colours correspond to the relative spindle abundance of each muscle.

| Supplementary Table 1. Percentage difference in muscle mass from Banks (2006) and those taken from the literature | | | |
| --- | --- | --- | --- |
| **Muscle** | **Average Mass Banks (g)** | **Average Mass Literature Review (g)** | **% Difference from Banks (2006)** |
| Arm | 34.0 ± 24.4 | 10.9 ± 4.4 | 60.9 ± 12.2 |
| Axial | 136.9 ± 86.6 | 79.2 ± 41.5 | 38.1 ± 13.5 |
| Foot | 16.1 ± 6.3 | 18.0 ± 5.6 | -26.6 ± 69.9 |
| Hand | 5.6 ± 5.1 | 2.9 ± 2.9 | 53.3 ± 28.6 |
| Hip | 205.7 ± 260.2 | 210.9 ± 299.1 | 19.4 ± 28.0 |
| Hyoid/Jaw | 7.1 ± 4.9 | 7.8 ± 10.3 | 4.4 ± 54.7 |
| Leg | 198.1 ± 170.1 | 209.9 ± 188.9 | -2.7 ± 27.5 |
| Neck | 17.6 ± 16.4 | 11.4 ± 12.4 | 34.6 ± 18.9 |
| Shoulder | 152.1 ± 113.3 | 120.2 ± 123.8 | 26.0 ± 23.4 |
| Mean ± SD | | | |
